# Pharmacogenetic-Guided Antidepressant Selection as an Opportunity for Interprofessional Collaboration: A Case Report

**DOI:** 10.3390/life11070673

**Published:** 2021-07-09

**Authors:** Céline K. Stäuble, Markus L. Lampert, Thorsten Mikoteit, Martin Hatzinger, Kurt E. Hersberger, Henriette E. Meyer zu Schwabedissen

**Affiliations:** 1 Biopharmacy, Department of Pharmaceutical Sciences, University of Basel, 4056 Basel, Switzerland; h.meyerzuschwabedissen@unibas.ch; 2 Pharmaceutical Care, Department of Pharmaceutical Sciences, University of Basel, 4001 Basel, Switzerland; markus.lampert@unibas.ch (M.L.L.); kurt.hersberger@unibas.ch (K.E.H.); 3 Institute of Hospital Pharmacy, Solothurner Spitäler, 4600 Olten, Switzerland; 4 Psychiatric Services Solothurn, Solothurner Spitäler and Department of Medicine, University of Basel, 4503 Solothurn, Switzerland; thorsten.mikoteit@spital.so.ch (T.M.); martin.hatzinger@spital.so.ch (M.H.)

**Keywords:** antidepressant drugs, depression, pharmacogenetics, psychiatry, pharmaceutical care, interprofessional relations, vortioxetine, CYP2D6, CYP2C19, ABCB1

## Abstract

In the herein reported case of a 42-year-old woman diagnosed with anxiety and depression, a long history of antidepressant ineffectiveness and adverse drug reactions was decisive for an in-depth medication review including pharmacogenetic panel testing. In detail, treatment attempts with paroxetine and escitalopram were ineffective and discontinued due to subjective gastrointestinal intolerance. Due to the worsening of the depression after the failed treatment attempts, admission to our clinic became necessary. Herein, owing to the collaboration of psychiatrists with clinical pharmacists, individualized incorporation of pharmacogenetic data into the process of antidepressant selection was enabled. We identified vortioxetine as a suitable therapeutic, namely for being most likely pharmacokinetically unaffected as predicted by pharmacogenetic panel testing and taking into account the current comedication, as well as for its favorable action profile. Herein, our collaborative effort proved to be successful and resulted in the patient’s depression remission and clinic discharge with the interprofessionally selected pharmacotherapy. This exemplary case not only highlights the potential benefits and challenges of pre-emptive pharmacogenetic testing in antidepressant prescription, but also proposes an approach on how to put pharmacogenetics into practice.

## 1. Background

Pharmacotherapy, in addition to behavioral therapy and others, is an important pillar in the treatment of major depressive disorder (MDD). Today, prescribing clinicians can choose from a wide range of marketed antidepressants. However, successful treatment of depression remains challenging and inter-individual differences in response to antidepressants are common. Indeed, around half of unipolar depressed patients do not respond to the first treatment attempt [1,2]. Moreover, the experience of serious adverse events under antidepressant pharmacotherapy and discontinuation due to intolerance of the same has been associated with therapy failure [2]. In particular, divergent levels of systemic drug exposure can cause inter-individual drug responses, leading to either toxicity in the case of supratherapeutic drug levels or ineffectiveness due to subtherapeutic drug levels. Apart from avoidable factors such as drug–drug or food–drug interactions and insufficient adherence, deviations in drug levels can also be caused by given predispositions, such as impaired renal or liver function, and, notably, genetics. In fact, many antidepressants are metabolized by highly polymorphic cytochromes P450 (CYP) including CYP2D6 and CYP2C19. For these enzymes, individuals can exhibit phenotypes with altered activity ranging from poor to ultrarapid metabolizers. Especially for CYP2D6 and CYP2C19, these phenotypes find their origin in the genetic make-up and can therefore be predicted by genotyping of associated single-nucleotide polymorphisms or copy number variations [3]. We have recently reported a case in which CYP genotypes might have substantially impaired antidepressant drug response over the years [4]. Moreover, the known influence of polymorphisms on antidepressant pharmacokinetics, toxicity and treatment response is already highlighted on numerous drug labels of marketed products [5]. Additionally, multiple guidelines with genotype-based recommendations for drug dosing and selection have been published and are currently available for tricyclic antidepressants and selective serotonin reuptake inhibitors [6,7]. Furthermore, the Swiss Society for Anxiety and Depression (SGAD) recommends genotyping of the P-glycoprotein (encoded by *ABCB1*) after experiencing antidepressant treatment failure [8]. The efflux transporter P-glycoprotein has an important gatekeeping role at the blood–brain barrier, where it extrudes xenobiotics and drug molecules including certain antidepressants. It is hypothesized that homozygous carriers of the wildtype allele may experience increased efflux of substrate antidepressants, leading to decreased drug levels within the central nervous system, which is their site of action. This theory is based on a limited number of clinical studies that linked certain *ABCB1* polymorphisms to antidepressant treatment response [9,10,11].

However, despite the already compiling evidence, especially for SSRIs and tricyclic antidepressants [6,7], pharmacogenetic (PGx) analysis is not yet routinely applied when prescribing these antidepressants. Underlying reasons are diverse and barriers to the implementation of PGx services include fragmentary evidence from prospective clinical trials, limited reimbursement from basic health insurance (which, in Switzerland, is currently only possible if clinical pharmacologists prescribe the specific testing), missing established procedures and, in general, a lack of education and experience among mental health care providers [12,13]. An approach to overcome some of these barriers, to efficiently enable individualized PGx information processing for antidepressant selection and dosing, might involve the interprofessional collaboration of psychiatrists and clinical pharmacists. The added value of an interdisciplinary approach concerning medication review in the psychiatry setting has been investigated before and was found to have a significant impact on the detection and solution of drug-related problems [14]. As described beforehand, pharmacogenetic predisposition might be a cause of drug-related problems such as adverse drug reactions and ineffectiveness. To illustrate the challenges and benefits of such an interdisciplinary PGx service, we herein report an exemplary case where individually interpreted PGx data were used in the course of collaborative decision-making on readjusting antidepressant pharmacotherapy.

## 2. Case Presentation

### 2.1. Clinical Case and Medication History

A 42-year-old female patient diagnosed with a generalized anxiety disorder (ICD-10 F41.1) and a recurrent depressive disorder (ICD-10 F33), without any other comorbidity diagnosed, entered our clinic for inpatient treatment due to acute mental decompensation manifested by reduced appetite, weight loss, abdominal pain without underlying somatic cause, sleeping disorder and lethargy. The recent deterioration in the patient’s condition was found to be multifactorial, inter alia caused by increasing familiar burden, recent therapy with childhood trauma processing and stress triggered by the COVID-19 pandemic. At admission, the current depressive episode without psychotic symptoms was rated as severe (ICD-10 F33.2), i.e., the rater-assessed 21-item Hamilton Rating Scale of Depression (HAM-D21) [15] yielded a score of 33 and the self-rating scale Beck Depression Inventory (BDI) [16] showed a score of 40. Prior outpatient treatment attempts included pharmacotherapy with paroxetine and escitalopram, both of which were discontinued due to subjective gastrointestinal intolerance and with insufficient therapeutic effect. As a result, the patient developed a strong fear of medication and potential adverse drug reactions, so that she refused a further therapeutic approach in the outpatient setting. At the clinic, an initial treatment attempt with pregabalin 25 mg daily was discontinued after only two days, upon the patient’s complaining of muscle cramps. Additionally, treatment with quetiapine was limited to a low-dose intake at night, due to the occurrence of daytime fatigue at higher dosage. Eventually, a therapy with agomelatine 50 mg at night was implemented and well tolerated. However, due to the limited effect of agomelatine monotherapy in the treatment of the underlying anxiety and the current severe depressive episode, a combination with escitalopram was introduced. With the help of a liquid formulation, a gradual dosage increase over the course of two weeks was attempted, due to the aforementioned subjective intolerance experienced in the past, under escitalopram dosages of up to 15 mg daily. At the present time, the patient tolerated a daily dosage of up to 10 mg escitalopram well. Meanwhile, laboratory parameters for liver and kidney function were assessed, revealing values in a normal range (e.g., serum creatinine, total bilirubin, ALAT and ASAT). However, the patient showed persisting unresponsiveness after almost 4 weeks of inpatient treatment. Therefore, and due to the known involvement of the polymorph CYP2C19 in escitalopram metabolism, the treating physician requested a pharmacogenetic consultation by clinical pharmacists of the hospital. This clinical pharmacy service includes a comprehensive medication review of the current medication as well as a semi-structured interview to gain information on the patient’s medication history and prior experiences with therapy failure and adverse drug reactions. At present, this pharmacogenetic consultation is part of an observational case study approved by the local ethics committee (ClinicalTrials.gov identifier: NCT04154553). Written informed consent for genetic testing and health data retrieval was collected from the patient prior to the intervention. Eventually, pharmacists classified the present case as potentially relevant in the context of pharmacogenetics and panel pharmacogenotyping was conducted from a buccal swab, applying the commercial service Stratipharm^®^ offered by humatrix AG (Pfungstadt, Germany). In their laboratory, the polymorphisms are determined by applying real-time PCR using the automated Life Technologies QuantStudio 12 k flex (Thermo Fisher, MA, USA) with the respective optimized and commercially available chemistry. Interpretation of the genotyping results identified the patient as CYP2C19 rapid metabolizer (RM, *17 heterozygous), CYP2D6 and CYP2B6 normal metabolizer (NM, *1 homozygous). Furthermore, the patient exhibited genetic variants associated with increased inducibility of CYP1A2 (*1F homozygous), and no variation in the *ABCB1* polymorphism rs2032583. Additionally, the analyzed *HTR2A* gene locus exhibited a homozygous variation for the rs7997012 polymorphism (Table 1).

### 2.2. Pharmacogenetic Data Interpretation

The gastrointestinal adverse drug reactions experienced in the past after the intake of escitalopram and paroxetine are frequently observed (1–10%) [17]. In the herein presented case, the underlying genetic profile, however, was not associated with an increased risk of adverse drug reactions due to the supratherapeutic drug levels of these substances. In the case of paroxetine, which is mainly metabolized by CYP2D6 with herein predicted normal activity (NM, *1 homozygous), treatment can be initiated with the usual recommended starting dose [6]. Escitalopram is extensively metabolized by CYP2C19, which, in the present case, was predicted with increased activity (RM, *17 heterozygous) and associated with an elevated risk of therapy failure [6]. Indeed, the patient did not respond to escitalopram after reintroducing it at our clinic. Nevertheless, side effects cannot be excluded per se. However, considering the fact that the reintroduction of escitalopram in the inpatient setting was well tolerated, a potential psychosomatic cause of the experienced gastrointestinal disorders might be discussed. Polychroniou et al. (2018) stated in their evaluation of treatment-naïve adults (n = 105) that escitalopram-associated side effects are dose-dependent [18]. Whether this also applies to the escitalopram re-exposure remains unclear. It seems noteworthy that there are data linking gastrointestinal distress during paroxetine and escitalopram intake to altered gut microbiota composition [19,20], but whether this also contributes to the disease symptoms remains to be further investigated.

Besides the gastrointestinal side effects, the antidepressants used previously, namely paroxetine, escitalopram and agomelatine, had not been effective. In the case of escitalopram, this most likely can be attributed to the increased activity of CYP2C19, through which escitalopram is extensively metabolized. Accordingly, current guidelines recommend consideration of an alternative antidepressant that is not predominantly metabolized by CYP2C19, due to the risk of inefficacy as a consequence of subtherapeutic drug levels (e.g., [6]). Furthermore, it seems noteworthy that the patient was a homozygous carrier of the CYP1A2 (*1F) variant, which is known to be linked to the enhanced inducibility of this particular CYP enzyme [21,22]. Together with the patient’s smoking status, this genetic profile could be linked to an increased degradation of agomelatine, which, when given as a monotherapy, indeed did not improve the patient’s depression. However, no guidelines for PGx-guided agomelatine selection and dosing are currently available. 

Additionally, other mechanisms than the CYP-related metabolism may have played a role in this individual’s medication history. One of these mechanisms may be the activity of the efflux transporter P-glycoprotein (encoded by *ABCB1*). The *ABCB1* rs232583 major allele variant has been associated with reduced therapy response in the treatment with substrates of this efflux transporter. Here, the transporter, which is known to be expressed in the blood–brain barrier, is assumed to limit brain entry, resulting in lower efficacy of centrally active molecules; these also include the molecules used in the herein reported patient, paroxetine and escitalopram [9,10,11]. It remains to be determined whether genetic variants such as the rs232583, which has been associated with the reduced efficacy of ABCB1 substrates, also influences the effect of P-glycoprotein as a determinant of oral bioavailability due to its apical expression in enterocytes. However, data supporting this notion are rather limited. In the context of antidepressants, which are known to modulate the serotonin homeostasis [23], the genetic profile of the serotonin receptor (*HTR2A*) was also evaluated within the herein applied commercial system by humatrix AG. Here, the patient exhibited the homozygous variant allele rs7997012, which has been linked with a decreased response to therapeutic interventions with es-/citalopram [24]. In this context, it seems to be noteworthy that the frequencies of the previously discussed genetic polymorphisms may vary across different ethnic populations and that, in this case, we are reporting a single patient of European descent.

Based on the analysis of the genetic profile and the patient’s medication history, taking into account the known contribution of the altered CYP2C19 and CYP1A2 to the metabolism as well as potentially ABCB1 to the transport of various antidepressants, the clinical pharmacist recommended the following substances for therapy optimization: vortioxetine, bupropion or venlafaxine. All of these are primarily metabolized by CYP enzymes with normal activity as predicted by panel pharmacogenotyping, i.e., vortioxetine and venlafaxine via CYP2D6 [25,26] and bupropion via CYP2B6 [27]. Moreover, these compounds exhibit slight differences in their pharmacodynamic profile, which, independent of the genotype, impacts the individual’s response. The physician decided together with the patient to change the antidepressant therapy to vortioxetine at week five of the hospitalization. This shared decision-making was supported on the one hand by the pharmacist’s reasoning that vortioxetine is primarily metabolized via the normally active CYP2D6, is not a relevant P-glycoprotein substrate [25] and would have no expected interaction with the patient’s current co-medication. On the other hand, vortioxetine had a suitable pharmacodynamic profile for the present case, i.e., mood-lifting and anxiety-relieving, favored by the psychiatrist. Thus, escitalopram and quetiapine were discontinued and vortioxetine 10 mg daily augmented with low-dose aripiprazole 2.5 mg daily was started instead, as an add-on to the already established agomelatine 50 mg daily. Notably, pharmacologic augmentation is a common strategy in the treatment of therapy-resistant MDD [28]. After five more weeks under treatment with the above-described regimen, the patient was discharged with remitted symptoms as evidenced by a HAM-D21 score of 4 (at admission: 33) and a BDI score of 7 (at admission: 40). 

## 3. Conclusions and Outlook

The interprofessional collaboration between psychiatrists and clinical pharmacists facilitated an individualized therapy approach with interpretation and incorporation of PGx data into the antidepressant selection process. Changing to an antidepressant with most likely unaffected pharmacokinetics as predicted by the genetic panel test and taking into account the current comedication and medication history, in combination with a favorable profile of action, was successful, as shown by good tolerability and remission of depression with the interprofessionally selected pharmacotherapy. It may be speculated that an early approach with PGx testing might have significantly reduced the patient’s burden as well as the duration of hospitalization. However, we are aware that we are reporting a single case, which does not allow for generalized conclusions, and the aforementioned hypotheses will have to be further tested in prospective studies [29]. Cases such as this support the notion that pre-emptive genotyping, or perhaps phenotyping, if available in a clinical setting [30], would be of great value for the patient and potentially cost-effective for the health care sector by enhancing the prescription of an effective pharmacotherapy at an early stage and thereby potentially reducing the duration and number of hospitalizations. However, at least in Switzerland, there is no clear or formal structure to support these advances. It is the aim of an ongoing research program to evaluate and establish these structures for an interprofessional collaboration of pharmacists (community and hospital) and the treating physicians [31]. 

## Figures and Tables

**Table 1:** Selected results of the panel pharmacogenotyping and phenotype interpretation thereof.

Gene	Variant *(Also Tested Variants in Gene Locus)*	Genotype	Predicted Phenotype
*CYP1A2*	rs762551 g.75041917C>A (in *1F) *(rs2069514)*	A/A	Increased inducibility
*CYP2B6*	*(rs8192709, rs28399499, rs3745274)*	WT ^3^, *1	Normal function (NM ^1^)
*CYP2C19*	rs12248560 g.4195C>T (in *17) *(rs4986893, rs4244285, rs28399504)*	C/T	Increased function (RM ^2^)
*CYP2D6*	*(CNV, rs35742686, rs3892097, rs5030655, rs5030867, rs5030865, rs5030656, rs1065852, rs201377835, rs28371706, rs59421388, rs28371725)*	WT ^3^, *1	Normal function (NM ^1^)
*ABCB1*	rs2032583 c.2685+49T>C *(rs1045642, rs1128503, rs2032582)*	T/T (WT ^3^)	Substance specific function
*HTR2A*	rs7997012 c.614-2211T>C *(rs6311, rs6313, rs9316233, rs6314)*	C/C	Substance specific function

^1^ NM: normal metabolizer; ^2^ RM: rapid metabolizer; ^3^ WT: wild type.

## Data Availability

The genetic data presented in this study are available on request from the corresponding author. The data are not publicly available for ethical and privacy reasons.
